# Data sharing in public health emergencies: a call to researchers

**DOI:** 10.2471/BLT.16.170860

**Published:** 2016-03-01

**Authors:** Christopher Dye, Kidist Bartolomeos, Vasee Moorthy, Marie Paule Kieny

**Affiliations:** a Department of Strategy, Policy & Information, World Health Organization, avenue Appia 20, Geneva 27, Switzerland.; b Department of Immunization, Vaccines & Biologicals, World Health Organization, Geneva, Switzerland.; c Health Systems and Innovation Cluster, World Health Organization, Geneva, Switzerland.

Data are the basis for public health action, and rapid data sharing is critical during an unfolding health emergency.^1^^,^^2^ The information disseminated through peer-reviewed journals and accompanying online data sets is vital for decision-makers.^1^

The deficiencies with existing data-sharing mechanisms, which were highlighted during the 2013–16 Ebola epidemic in west Africa, have brought the question of data access to the forefront of the global health agenda.^2^ In September 2015, agreement was reached on the need for open sharing of data and results, especially in public health emergencies.^3^ Subsequently, following published expressions of support by its members, the International Committee of Medical Journal Editors (ICMJE) have explicitly confirmed that pre-publication dissemination of information critical to public health will not prejudice journal publication in the context of a public health emergency declared by WHO.^4^ While efforts so far have focused on results from clinical trials, and on making full accompanying data sets available at the time of publication, there are further opportunities to expand access to information from observational studies, operational research, routine surveillance and the monitoring of disease control programmes.

To improve timely access to data in the context of a public health emergency, the *Bulletin of the World Health Organization* will implement a new data sharing and reporting protocol. The protocol is established specifically to address the data gap that exists in responding to the current Zika virus epidemic, and will apply in the first instance only to articles submitted in the context of this outbreak.

On submission to the *Bulletin*, all research manuscripts relevant to the Zika epidemic will be assigned a digital object identifier and posted online in the “Zika Open” collection within 24 hours while undergoing peer review. The data in these papers will thus be attributed to the authors while being freely available for reader scrutiny and unrestricted use, distribution and reproduction in any medium, provided that the original work is properly cited as indicated by the Creative Commons Attribution 3.0 Intergovernmental Organizations license (CC BY IGO 3.0)^5^. Should a paper be accepted by the *Bulletin* following peer review, this open access review period will be reported in the final publication. In the event that a paper does not survive peer review, and given the rapidly evolving knowledge basis on this disease, authors will be free to seek publication elsewhere. If the authors of any paper posted with the *Bulletin* in this context are unable to obtain acceptance with a suitable journal, WHO undertakes to publish these papers in its institutional repository as citable working papers, independently of the *Bulletin*. This early access to research manuscripts at WHO builds on examples of other rapid information access platforms such as PROMED and F1000Research.^6^^,^^7^

Given the number and complexity of unanswered questions on the mechanisms and consequences of Zika infection and associated disease, our goal is to encourage all researchers to share their data as quickly and widely as possible. With this protocol for immediate online posting, we are providing another means to achieve immediate global access to relevant data. Researchers can thus share their data while meeting their need to retain authorship, achieve precedence, and to put their research on public record. We are pleased to announce that the first paper to which this protocol applies is now available online.^8^
